# Divergence between the high rate of p53 mutations in skin carcinomas and the low prevalence of anti-p53 antibodies

**DOI:** 10.1054/bjoc.2001.2185

**Published:** 2001-12

**Authors:** C Moch, A Moysan, R Lubin, P de La Salmonière, N Soufir, F Galisson, C Vilmer, E Venutolo, F Le Pelletier, A Janin, N Basset-Séguin

**Affiliations:** Unité INSERM U532, Institut de Recherche sur la Peau, Hôpital Saint-Louis, Paris, France; Pharmacell, Paris, France; Département de Biostatistique et Informatique Médicale, Hôpital Saint-Louis, Paris, France; Laboratoire de Recherche de Pathologie UPRES EA2378, Hôpital Saint-Louis, Paris, France

**Keywords:** skin tumours, basal cell carcinomas, squamous cell carcinoma, anti-p53 antibodies, tumoural markers

## Abstract

Circulating anti-p53 antibodies have been described and used as tumoural markers in patients with various cancers and strongly correlate with the p53 mutated status of the tumours. No study has yet looked at the prevalence of such antibodies in skin carcinoma patients although these tumours have been shown to be frequently *p53* mutated. Most skin carcinoma can be diagnosed by examination or biopsy, but aggressive, recurrent and/or non-surgical cases' follow up would be helped by a biological marker of residual disease. We performed a prospective study looking at the prevalence of anti-p53 antibodies using an ELISA technique in a series of 105 skin carcinoma patients in comparison with a sex- and age-matched control skin carcinoma-free group (*n* = 130). Additionally, p53 accumulation was studied by immunohistochemistry to confirm p53 protein altered expression in a sample of tumours. Anti-p53 antibodies were detected in 2.9% of the cases, with a higher prevalence in patients suffering from the more aggressive squamous cell type (SCC) of skin carcinoma (8%) than for the more common and slowly growing basal cell carcinoma type or BCC (1.5%). p53 protein stabilization could be confirmed in 80% of tumours studied by IHC. This low level of anti-p53 antibody detection contrasts with the high rate of p53 mutations reported in these tumours. This observation shows that the anti-p53 humoral response is a complex and tissue-specific mechanism. © 2001 Cancer Research Campaign http://www.bjcancer.com


*British Journal of Cancer* (2001) **85**(12), 1883–1886
© 2001 Cancer Research Campaign
doi: 10.1054/ bjoc.2001.2185, available online at http://www.idealibrary.com on IDEAL

### Divergence between the high rate of p53 mutations in skin carcinomas and the low prevalence of anti-p53 antibodies

**C Moch[1], A Moysan[1], R Lubin[2], P de La Salmonière[3], N Soufir[1], F Galisson[1], C Vilmer[1], E Venutolo[1], F Le Pelletier[4], A Janin[4] and N Basset-Séguin[1]**

[1]Unité INSERM U532, Institut de Recherche sur la Peau, Hôpital Saint-Louis, Paris, France; [2]Pharmacell, Paris, France; [3]Département de Biostatistique et Informatique Médicale, Hôpital Saint-Louis, Paris, France; [4]Laboratoire de Recherche de Pathologie UPRES EA2378, Hôpital Saint-Louis, Paris, France

**Summary** Circulating anti-p53 antibodies have been described and used as tumoural markers in patients with various cancers and strongly correlate with the p53 mutated status of the tumours. No study has yet looked at the prevalence of such antibodies in skin carcinoma patients although these tumours have been shown to be frequently *p53* mutated. Most skin carcinoma can be diagnosed by examination or biopsy, but aggressive, recurrent and/or non-surgical cases' follow up would be helped by a biological marker of residual disease. We performed a prospective study looking at the prevalence of anti-p53 antibodies using an ELISA technique in a series of 105 skin carcinoma patients in comparison with a sex- and age-matched control skin carcinoma-free group ($n$ = 130). Additionally, p53 accumulation was studied by immunohistochemistry to confirm p53 protein altered expression in a sample of tumours. Anti-p53 antibodies were detected in 2.9% of the cases, with a higher prevalence in patients suffering from the more aggressive squamous cell type (SCC) of skin carcinoma (8%) than for the more common and slowly growing basal cell carcinoma type or BCC (1.5%). p53 protein stabilization could be confirmed in 80% of tumours studied by IHC. This low level of anti-p53 antibody detection contrasts with the high rate of p53 mutations reported in these tumours. This observation shows that the anti-p53 humoral response is a complex and tissue-specific mechanism. © 2001 Cancer Research Campaign http://www.bjcancer.com

**Keywords**: skin tumours; basal cell carcinomas; squamous cell carcinoma; anti-p53 antibodies; tumoural markers

The p53 protein is encoded by the *p53* tumour suppressor gene and plays a crucial role in the protection of our cells against a genotoxic stress. This nuclear phosphoprotein is expressed at a very low level and is hardly detectable in normal cells. Alteration of the *p53* tumour suppressor gene is the most frequent genetic event found in human cancer and leads to loss of function and accumulation of the inactive p53 protein in malignant cells (Hollstein et al, 1991).

Human anti-p53 antibodies have been described as early as 1982 (Crawford et al, 1982). These anti-p53 antibodies are highly specific of malignant diseases and their detection correlates with inactivation of the *p53* gene in tumoural cells (Collet et al, 1997; Hammel et al, 1999). A number of studies have looked at their usefulness as a marker of malignancy. These studies have shown that anti-p53 antibodies were rarely detected in healthy donors and patients with benign diseases and that in cancer patients their prevalence varied from 2 to 25% according to the tumour studied (Schlichtholz et al, 1992; Lubin et al, 1995; Hammel et al, 1997).

Anti-p53 antibodies have appeared as complementary tumoural markers in the follow-up of patients with colorectal or lung cancers (Lubin et al, 1995; Zalcman et al, 1998). In these cancers, anti-p53 antibodies, when positive, decrease rapidly in the context of therapeutic control of the disease and increase before the metastatic progression can be diagnosed by conventional techniques. Furthermore, clinical studies have shown that anti-p53 antibodies are an independent prognostic factor of short survival in breast cancer and head and neck epidermoid carcinoma (Peyrat et al, 1995; Bourhis et al, 1996; Kaur et al, 1997). More importantly, anti-p53 antibodies have proven to be predictive markers for the detection of a malignant tumour in high-risk patients such as in chronic bronchitis patients for the early detection of lung carcinoma (Lubin et al, 1995) or in dysplastic lesions of the oral cavity for the further development of a carcinoma at the same location (Kaur et al, 1997). All these results strongly support the hypothesis that anti-p53 antibodies are a new tumoural marker of great potential interest in clinical oncology.

Because of the high frequency of *p53* mutations in human skin carcinomas (Basset-Séguin et al, 1994) and their occurrence as early as in chronically sun-exposed skin (Ren et al, 1996), we hypothesized that these tumours could be a perfect target for anti-p53 antibody production. Most skin carcinomas can be detected by inspection and biopsy. However, in patients with aggressive tumours, clinical and radiological follow up after surgical treatment or chemotherapy is often difficult because of local tissue remodelling. Additionally, tumours on highly damaged skin in patients having multiple and recurrent lesions are not easy to diagnose. To our knowledge no data are available in the litterature on the prevalence of anti-p53 antibodies and their usefulness as a tumoral marker in skin carcinoma. To determine whether anti-p53 antibodies could be a relevant tumoural marker for skin carcinoma, we conducted a prospective study to determine whether anti-p53 antibodies were more frequent in skin carcinomas, in

*Received 4 June 2001*
*Revised 19 September 2001*
*Accepted 20 September 2001*

*Correspondence to:* N Basset-Séguin particular basal cell carcinoma (BCC) and squamous cell carcinoma (SCC), than in skin-carcinoma-free patients. Additionally, in order to confirm *p53* gene alteration, which usually correlates with detection of anti-p53 antibodies, we checked for p53 protein stabilization in a sample of these tumours by immunohistochemistry.

### PATIENTS AND METHODS

### Patients

Consecutive patients consulting at the Hôpital Saint-Louis for skin carcinoma were prospectively enrolled in the study with the following inclusion criteria: age > 18 years, histologically proven skin carcinoma (BCC, SCC, adnexal tumours, Bowen, actinic keratosis (AK)), no surgery, chemotherapy and/or radiotherapy yet started, patient's consent for a single blood sampling. For each patient the following data were recorded independently of the serological status to p53: age, gender, histological subtype, number of tumours, existence of a genodermatosis predisposing to cancer. Controls were chosen from patients coming in the dermatology day-care unit during the same study period. Their skin carcinoma-free status was verified by patient's history and complete physical examination. These controls were matched with the cases according to gender and age.

From September 1997 to January 1999, 105 patients were enrolled in the study. Main patients' characteristics were age: median 70 years (range 26–98 years), male gender 49.5% ($n$ = 52). The type and number of tumours are listed in Table 1. In the control group ($n$ = 130) the main characteristics were: age – median 63 years (range 25–97), male gender 48% ($n$ = 63). The diagnoses of the control group are shown in Table 2.

**Table 1** Skin tumour type in our series of patients

| Tumour type | Unique | Multiple |
|---|---|---|
| BCC | 39 | 29 |
| SCC | 22 | 3 |
| BCC + SCC | – | 2 |
| Bowen | 3 | – |
| Sweat gland carcinoma | 1 | – |
| Actinic keratosis | – | 2 |
| NK | 4 | – |
| Total | 69 | 36 |

BCC: patients with basal cell carcinoma alone; SCC: patients with squamous cell carcinoma alone; BCC + SSC: patients with both SCC and BCC; NK: skin carcinoma type not known.

**Table 2** Diagnoses of the control group

| Dermatoses | Number of patients analysed |
|---|---|
| Allergic dermatitis | 14 |
| Auto-immune diseases | 20 |
| Prurigo | 19 |
| Cutaneous lymphoma | 21 |
| Psoriasis, neutrophilic dermatosis | 6 |
| Lichen | 2 |
| Vasculitis | 2 |
| Sarcoidosis | 6 |
| Benign lymphocytic infiltrate | 13 |
| Other | 20 |
| Not determined | 7 |
| Total | 130 |

### Detection of p53 autoantibodies

Detection of anti-p53 antibodies in human sera was made by using a commercially available enzyme-linked immunoabsorbent assay kit (anti-p53 ELISA, Pharmacell, France). Sera were diluted to 1/100 and incubated for 1 hour in the microplates. After washing, goat anti-human IgG antibody conjugated with peroxidase was added for 1 hour. Next, the substrate 3, 3′, 5, 5′ tetramethylbenzidine (TMB) was added for 10 min and the enzymatic process was halted by adding 2N sulfuric acid. Light absorption was measured at 450 nm using a phosphospectrometer. Because human sera may give rise to variable background signals, each sample was tested simultaneously in 2 distinct wells. One well was coated with recombinant wild-type human p53 protein to detect specific anti-p53 antibodies. The other well was coated with control proteins to detect non-specific interactions. The presence of anti-p53 antibodies in the sample was determined according to the manufacturer for 2 parameters at threshold levels, allowing evaluation of the sample compared with controls.

### Immunohistochemistry

Skin samples fixed in 10% formalin, were deparaffinized, and stained after antigen retrieval in a microwave oven 15 m, at 450 w in tris buffer pH 7.3 with monoclonal antibody D-07 (Dako, code M7001), which reacts with both wild-type and mutant forms of the human p53 protein, at a dilution of 1:50, and revealed by avidin–biotin-coupled immunoperoxidase staining method and diaminobenzidin. A positive control (a p53 mutated tumour) was always added. Staining was nuclear and was negative when omiting the primary antibody. A tumour was found positive if at least 30% of nuclei were stained. Staining intensity was graded + if nuclei were light brown, ++ if nuclei were brown and +++ if nuclei were dark brown. Mayer's haematoxylin was used for counterstaining. Slides were read by 2 pathologists independently.

### Statistical analysis

Comparisons between groups were performed with Fisher's exact test for categorical variables; 95% confidence intervals (95% CI) of percentages were calculated with the exact binomial method. 2-sided tests were computed, and $P$ values of 0.05 or less were considered statistically significant. The SAS software (SAS Institute, Cary, NC) was used.

### RESULTS

### Prevalence of anti-p53 antibodies

The estimated prevalence of anti-p53 antibodies among patients with skin carcinomas is shown in Table 3.

In the control group, the estimated prevalence of anti-p53 antibodies was 2/130 (0.015, 95% CI: 0–0.05).

There was no significant difference of anti-p53 antibodies prevalence between skin cancer patients and controls ($P$ = 0.66); BCC patients and controls ($P$ = 1.00); SCC patients (8%) and controls (1.5%) ($P$ = 0.12); SCC patients and BCC patients ($P$ = 0.17).

*© 2001 Cancer Research Campaign*

**Table 3** Prevalence of circulating anti-p53 antibodies and tumoural p53 immunoreactivity in our series of patients

| Tumour type | Anti-p53 antibodies | Tumour p53 + (IHC) |
|---|---|---|
| BCC | 1.5% (1/68) 95% CI: (0–0.08) | 85% (22/26) |
| SCC | 8% (2/25) 95% CI: (0.01–0.26) | 62% (5/8) |
| BCC + SCC | 0% (0/2) | ND |
| Bowen | 0% (0/3) | 100% (1/1) |
| Adnexal carc | 0% (0/1) | ND |
| AK | 0% (0/2) | ND |
| NK | 0% (0/4) | ND |
| Total tumours | 2.9% (3/105) | 80% (28/35) |

NK: not known; Adnexal carc: adnexal carcinoma; AK: actinic keratosis; ND: not determined; IHC: immunohistochemistry. 95% confidence intervals (95% CI) are given for BCC and SCC alone.

Positive sera levels were: 1.31, 4.4 and 5.23 with a threshold of positivity of 1.1.

### p53 stabilization in the skin tumour

Among the 105 patients tested for anti-p53 antibody, a sample of 35 tumours (26/39 BCC and 8/25 SCC and 1 Bowen) was randomly selected and analysed for p53 stabilization by immunohistochemistry in order to check for evidence of p53 protein accumulation. Among the tumours analysed 80% ($n$ = 28/35) showed positive nuclear staining within the tumoural nest. All were graded as ++ or +++. Histological types of p53-positive tumours are shown in Table 3. Among the 3 patients with circulating anti-p53 antibodies, one had a p53-positive tumour, one had a negative tumour and the third one could not be analysed.

### DISCUSSION

We estimated in this controlled prospective study the prevalence of circulating anti-p53 antibodies in skin carcinoma patients. Surprinsingly the high rate of p53 mutation reported by us and others for these tumours contrasted with the low prevalence of anti-p53 antibodies. This paradox is a rare phenomenon showing the complexity of the anti-p53 humoral response. Clearly, anti-p53 antibodies can not serve as early indicators for skin cancer-prone high-risk patients. However, anti-p53 antibody prevalence was higher in the most aggressive forms of SCC which suggest then when positive, these antibodies could serve to follow rare difficult non-surgical SCC cases during the course of therapy.

Circulating anti-p53 antibodies have been shown to be useful as prognostic and predictive tumoural markers in a number of human neoplasms, in which the p53 protein is accumulated. The prevalence of anti-p53 antibodies varies according to the tumour group analysed: 24–25% in colon or lung cancer, 12–19% in breast, bladder and pancreas cancer, and 3–4% in leukaemia or thyroid carcinomas. The prevalence of anti-p53 antibodies varies also with the detection method used, as immunofluorometry detection yields lower prevalences than the ELISA technique in all the studied tumours but with the same proportional differences.

In our series of patients the prevalence of anti-p53 antibodies was low (2.9%). This can not be due to a technical artefact as the ELISA assay that we used is the one commonly performed in many previously published studies. This low prevalence contrasted with the high rate of p53 mutations classically reported for these tumours (Basset-Séguin et al, 1994). A recent review of over 130 papers published in the field since 1992 showed a strong correlation between anti-p53 antibody detection and the presence of p53 mutation in the tumour in most human cancers (Soussi, 2000). One exception to this is the observation of a poor anti-p53 humoral response in gliomas in which p53 mutation rate is high (Rainov et al, 1995). Hypothetical explanation for this paradox is the immune privilege of the brain not allowing this humoral response or an inefficient antigen presentation due to the brain barrier or even a locally non-effective immune response. Additionally dexamethasone intake reported for 70% of the glioma patients before serum collection could have impaired the patient's immune capacity.

To check if the low prevalence of anti-p53 antibodies in our skin carcinoma patients was not due to a low prevalence of *p53* gene alteration, we looked for p53 protein accumulation by immunohistochemistry in a randomly selected subgroup of 35 patients. Elevated levels of p53 protein were detected in 80% of the tumours analysed which confirmed the frequent alteration of this pathway classically reported in skin carcinomas. One patient with circulating anti-p53 antibodies was found to have an accumulation of the P53 protein in its tumour. Another one was found negative but that does not mean that its tumour was not p53-mutated as mutation leading to protein conformational changes or stop mutation can be associated with lack of p53 protein accumulation. The third patient could not be evaluated for immunohistochemistry.

The differences in anti-p53 antibody prevalences among patients with p53-mutated tumours could be influenced by several factors.

1. p53 mutation location: although initially suggested, it is unlikely as a recent a compilation of serological analyses performed in association with molecular analysis has shown that p53 mutation do not differ between patients with or without anti-p53 antibodies (Soussi, 2000).
2. Tumour location: interestingly more than 80% of skin carcinomas are localized in sun-exposed skin and it is well known that sun-exposure has local as well as systemic immunosuppressive properties by affecting cytokine production and the number and capacity of antigen-presenting cells (Beissert and Schwarz, 1999). Thus we could hypothesize that sun-exposed human skin fails to elicit an efficient anti-p53 humoral response. Our patients were not immunocompromised but most of their tumour located in highly sun exposed skin. Indeed in our series, only one patient with circulating anti-p53 antibodies had a lesion on highly sun-exposed skin (forehead) and the 2 others had a tumour on moderately sun-exposed skin (back) or non-sun-exposed skin (vulva). In another UV-related tumour, melanoma, in which the p53 protein is frequently found accumulated but without concomitant mutation of the gene, a very small percentage (2%) of anti-p53 antibodies have also been detected (Angelopoulou, 1994).
3. Tumour thickness and tumoural mass could also be responsible for the poor immunization against p53 as skin tumours are often of small size. However, 3 of our patients suffered from Gorlin's syndrome, a genodermatosis predisposing to the development of numerous BCC, and beared between 30 and > 100 tumours which represent a large overall tumoural mass and had no anti-p53 antibodies.

To determine whether anti-p53 antibody prevalence differed between skin carcinoma and skin carcinoma-free patients recruited in a dermatology department, we estimated the anti-p53 antibodies prevalence in a control group including mostly chronic inflammatory

© 2001 Cancer Research Campaign dermatoses. Circulating anti-p53 antibodies were found in 2 out of 130 patients (1.5%). In the literature, the prevalence of anti-p53 antibodies in healthy donors has been shown to be 0.5% and in non-tumoural human pathology 1% (Schlichtholz et al, 1992; Lubin et al, 1995; Hammel et al, 1997). It is noteworthy that most studies on p53 antibodies in human tumours have not been performed with a tumour-free control group. Additionally, it has been shown that in non-tumoural inflammatory diseases such as rhumatoid arthritis, p53 accumulation could be found in the synovium (Firestein et al, 1996). The 2 skin carcinoma-free patients found to have anti-p53 antibodies were diagnosed as rheumatoid purpura and chronic urticaria.

In conclusion this is the first study reporting the prevalence of anti-p53 antibodies in skin carcinoma. Surprisingly, these frequently *p53*-mutated skin tumours are associated with a low prevalence of anti-p53 antibodies. UV-induced immunosuppression could explain in part this low prevalence. The highest prevalence of anti-p53 antibodies was observed in SCC patients compared to BCC patients or controls. These results suggest that these antibodies can not be used routinely in skin carcinoma patients, however, in difficult SCC cases, they may be helpful to follow response to therapy.

### ACKNOWLEDGEMENTS

This work was supported by INSERM, AP-HP Grant and GEFLUC.

### REFERENCES

Angelopoulou K, Diamandis EP, Sutherland DJA, Kellen JA and Bunting PS (1994) Prevalence of serum antibodies against the p53 tumor suppressor gene protein in various cancers. *Int J Cancer* **58**: 480–487

Basset-Séguin N, Molés JP, Mils V, Dereure O and Guilhou JJ (1994) TP53 tumor suppressor gene and skin carcinogenesis. *J Invest Dermatol* **103**: 102S–106S

Beissert S and Schwarz T (1999) Mechanism involved in ultraviolet light-induced immunosuppression. *J Invest Dermatol Symp Proceed* **4**: 61–64

Bourhis J, Lubin R, Roche B, Koscienly S, Bosq J, Dubois I, Talbot M, Marandas P, Schwaab G, Wibault P, Luboinski B, Eschwege F and Soussi T (1996) Analysis of p53 serum antibodies in patients with head and neck squamous cell carcinoma. *J Nat Cancer Inst* **88**: 1228–1233

Collet B, Raoul JL, Le Berre N, Heresbach D, Meritte H, Quillien V and de Certaines JD (1997) Serum anti-p53 antibodies in patients with squamous cell carcinoma of the esophagous: Comparison with p53 alterations and lymph node invasion. *Int J Oncol* **11**: 617–621

Crawford LV, Pim DC and Bulbrook RD (1982) Detection of antibodies against the cellular protein p53 in sera from patients with breast cancer. *Int J Cancer* **30**: 403–408

Firestein GS, Nguyen K, Aupperle KR, Yeo M, Boyle DL and Zvaifler N (1996) Apoptosis in rhumatoid arthritis. p53 overexpression in rheumatoid arthritis synovium. *Am J Pathol* **149**: 2143–2151

Hammel P, Boissier B, Chaumette MT, Piedbois P, Rotman N, Kouyoumdjian JC, Lubin R, Delchier JC and Soussi T (1997) Detection and monitoring of serum p53 antibodies in patients with colorectal cancer. *Gut* **40**: 356–361

Hammel P, Leroy-Viard K, Chaumette MT, Villaudy J, Falzone MC, Rouillard D, Hamelin R, Boissier B and Remvikos Y (1999) Correlations between p53-protein accumulation, serum antibodies and gene mutation in colorectal cancer. *Int J Cancer* **81**: 712–718

Hollstein M, Sidransky D, Vogelstein B and Harris C (1991) p53 mutations in human cancers. *Science* **253**: 49–53

Kaur J, Srivastava A and Ralhan R (1997) Serum p53 antibodies in patients with oral lesions: correlation with p53/HSP70 complexes. *Int J Cancer (Pred Oncol)* **74**: 609–613

Lubin R, Zalcman G, Bouchet L, Trédaniel J, Legros Y, Cazals D, Hirsch A and Soussi T (1995) Serum p53 antibodies as early markers of lung cancer. *Nat Med* **1**: 701–702

Peyrat JP, Bonneterre J, Lubin R, Vanlemmens L, Fournier J and Soussi (1995) Prognostic significance of circulating p53 antibodies in patients undergoing surgery for locoregional breast cancer. *Lancet* **345**: 621–622

Rainov, NG, Dobberstein KU, Fittkau M, Bahn H, Holzausen HJ, Gantchev L and Burkert W (1995) Absence of p53 antibodies in sera from glioma patients. *Clin Cancer Res* **1**: 775–781

Ren ZP, Hedrum A, Ponten F, Nister M, Ahmadian A, Lundeberg J, Uhlen M and Ponten J (1996) Human epidermal cancer and accompanying precursors have identical p53 mutations different from p53 mutations in adjacent areas of clonally expanded non-neoplasic keratinocytes. *Oncogene* **12**: 765–773

Schlichtholz B, Legros Y, Gillet D, Gaillard C, Marty M, Lane D, Calvo F and Soussi T (1992) The immune response to p53 in breast cancer patients is directed against immunodominant epitopes unrelated to the mutational hot spot. *Cancer Res* **52**: 6380–6384

Soussi T (2000) p53 antibodies in the sera of patients with various types of cancer: A Review. *Cancer Research* **60**: 1777–1788

Zalcman G, Schlichtholz B, Trédaniel J, Urban T, Lubin R, Dubois I, Milleron B, Hirsch A and Soussi T (1998) Monitoring of p53 autoantibodies in lung cancer during therapy: relationship to response to treatment *Clin Can Res* **4**: 1359–1366

 *© 2001 Cancer Research Campaign*
